# Oxidative stress in breast cancer after chemotherapy

**DOI:** 10.6026/973206300181141

**Published:** 2022-12-31

**Authors:** Tejaswi Pullakanam Sri Phani, Murugan Mannangatti, Ramakrishna Nekkala, Venkata Madhavi Bellala, Ravi Shankar Bellala, Vijayalakshmi Payala

**Affiliations:** 1 Department of Biochemistry, Aarupadai Veedu Medical College and Hospital, Vinayaka Mission's Research foundation (VMRF-DU), Pondicherry, Tamilnadu, India; 2 Department of Biochemistry, AarupadaiVeedu Medical College and Hospital, Vinayaka Mission's Research foundation (VMRF-DU), Pondicherry, Tamilnadu, India; 3 Department of Biochemistry, Gayatri Vidya Parishad Institute of Health Care and Medical Technology, Marikavalasa, Visakhapatnam, Andhra Pradesh, India; 4 Department of Pathology, GITAM Institute of Medical Sciences and Research, GITAM Deemed to be University, Visakhapatnam, Andhra Pradesh, India; 5 Clinical Oncology Division, Omega Hospital, Visakhapatnam, Andhra Pradesh, India; 6 Department of Microbiology, GITAM Institute of Medical Sciences and Research, GITAM Deemed to be University, Visakhapatnam-530045, Andhra Pradesh, India

**Keywords:** Breast cancer, chemotherapy, malondialdehyde, oxidative stress, total antioxidant status

## Abstract

It is of interest to evaluate the influence of breast cancer on oxidative stress, liver function tests, renal biomarkers, action of doxorubicin, cyclophosphamide and paclitaxel (AC-T) in the treatment and mechanism over altering the measured markers in breast cancer. Sixty histopathological confirmed cases of female patients suffering with breast carcinoma from the Department of Oncology at Omega Cancer Hospital, Visakhapatnam were included in the study. The investigation was performed in 3 groups: a control group containing 30 healthy females of similar age, 30 breast cancer patients without treatment and 30 patients receiving treatment with anticancer combination drugs AC-T. The venous blood samples from both controls and patients were measured for total antioxidant status (TAS), nitric oxide (NO), malondialdehyde, alanine aminotransferase, aspartate aminotransferase, and blood urea, serum creatinine. One-way ANOVA and Tukey-Kramer multiple comparisons post-test were applied as statistical analysis tools through SPPS software version 20.0. P<0.05 was regarded as significant. According to the findings, higher stages of breast cancer were linked to considerable increase in oxidative stress markers during AC-T treatment. The findings of the study revealed that oxidative stress is linked to breast cancer, and that chemotherapy exacerbates this oxidative stress, causing damage to a variety of cellular targets. Monitoring serum oxidative stress markers may aid in the evaluation of chemotherapy effects in breast cancer patients. According to our findings, AC-T chemotherapy will elevate malondialdehyde, a lipid peroxidation marker, and lowers the total antioxidant status.

## Background:

Human mortality rate is increasing day-by-day globally, though many sources are there, one of the foremost reason is cancer [1-2]. The frequent malignancy identified in women is Breast carcinoma [3-4]. Breast cancer has a complex etiology and the threat rises with menarche at early age, menopause at late stages, late pregnancy age, overweight, solid breast tissue, using oral contraceptive pills, treatments involving replacement of harmones, alcoholism, hereditary, tobacco use, lactation, diet, and a previous history of any non-malignant breast disorders [5, 6]. Breast cancer susceptibility and development have been related to a several genes, involving BRCA1 and BRCA2, HER2/neu, and p53 [7]. If there is a disparity occurs in between formation of oxygen free-radicals and antioxidant scavenging, it is known as oxidative stress, which plays a critical part in the origin, development, and dissemination of carcinoma of breast. Generationof high oxygen free radicals may induce oxidative mutilation to biomolecules, leading to mutagenesis, lipid peroxidation, and carcinogenesis [8, 9]. Antioxidants are oxidative stress defense mechanisms that eliminate reactive species and maintain redox balance [10]. By neutralizing free radicals, the antioxidant defense systems of cells both (enzymatic and non-enzymatic) will avoid the oxidant-mediated damage to the macromolecules including lipids, protein, and DNA [11-12]. The metabolic by-products of electron transport chain complexes I and III of mitochondria are reactive oxygen species (ROS) like O-2radical, OH- radical, and H2O2 [13]. The principal targets of ROS are polyunsaturated fatty acids (PUFA) and certain proteins integrated in cellular membrane [14]. Nitric oxide (NO), is well established to involve in the carcinogenesis of breast. Damage to DNA caused by oxidative stress and NO encompasses mutagenic changes in the cells and plays a pivotal role the development of cancer and ageing [15]. Formetastatic breast carcinoma, adjuvant chemotherapy a systemic treatment used after the tumour was surgically removed [4]. Cytotoxic chemotherapy medicines kill cancer cells by causing them to undergo apoptosis. Many earlier research works have revealed that antineoplastic medications can kill the tumour cells by causing apoptosis, which is accomplished, at least in part, by producing reactive oxygen species in the cells [16-18]. Anthracyclines have been among the most potent anticancer medications ever devised [19]. Majority of breast carcinoma patients under metastatic stage will receive a combination of anticancer drugs, such as doxorubicin and cyclophosphamide (AC-T), followed by paclitaxel [20]. Paclitaxel, a taxanes-related antineoplastic medication that affects microtubule stability, is a frequently used chemotherapeutic agent in different cancer types including breast cancer [21-22]. Hence the present investigation was mainly designed and implemented to evaluate the influence of breast cancer on oxidative stress, liver function tests, renal biomarkers, and also the action of doxorubicin, cyclophosphamide and paclitaxel (AC-T) in the treatment and mechanism over altering the measured markers in breast cancer.

## Materials and Methods:

Sixty female breast cancer patients from the Department of Oncology, Omega Cancer Hospital, Visakhapatnam were included in the study and it was approved by the Institutional Ethical Committee. The tumours were histo pathologically diagnosed, in the majority of instances as invasive ductal and invasive lobular carcinoma with stage II and stage III, and a few other forms are listed in Table 1(see PDF). The patients were fall in the age group between 30-75 years. The investigation was performed in 3 groups: a control group containing 30 healthy females of similar age, 30 breast cancer patients without treatment and 30 patients receiving treatment with anticancer combination drugs AC-T.AC-T Adriamycin/doxorubicin, cyclophosphamide and paclitaxel) mostly received 2 AC-T cycles (Doxorubicin- 60 mg/m2,Cyclophosphamide-600 mg/m2 and followed by Paclitaxel -175mg/m2). Patients with diabetes mellitus, high blood pressure, myocardial infarction, renal failure, pancreatic ailments, lung disease, infectious diseases, HIV/AIDS, allergic diseases, autoimmune diseases, and other malignancies, as well as patients, who had previously received radiation for any cancer, were excluded in the current study. Previous medical history from all the study subjects were collected, they were physically examined, and laboratory tests were performed.7ml of fasting Venous Blood was collected from the Normal control group and patients and serum was used to quantify the levels of catalase, malondialdehyde, nitric oxide (NO), total antioxidant status, blood urea, serum creatinine, alanine aminotransferase and aspartate aminotransferase.

## Methods:

Nitric oxide (NO) measurement of serum sample was performed by Griess method [23]. Lipid peroxide (malondialdehyde) was estimated by thiobarbituric acid reactive substances (TBARS) method [24]. Catalase assay through Continuous Spectrophotometric rate determination method was completed. Ferric reducing antioxidant power (FRAP) assay by Spectrophotometric method was used for Total antioxidant status determination [25].Liver enzymes like alanine aminotransferase (ALT) and aspartate aminotransferase (AST) were investigated based on the colorimetric estimation of pyruvate hydrazone and oxaloacetateat 546 nm. Serum creatinine and blood urea were measured using the Burtis and Ashwood method [26].

##  Statistical analysis:

One-way ANOVA and Tukey-Kramer multiple comparisons post-test were applied as statistical analysis tools through SPPS software version 20.0. P <0.05 was regarded as significant. Data was represented in the form of tables and figures as Mean ± SEM.

## Results:

The classification of breast cancer patients enrolled in the present study was shown in Table 1(see PDF).Majority of the patients were left breast affected individuals 33(55%). Most of them have invasive ductal carcinoma 40(66.6%) and express the receptor ER+ profoundly 46(76.6%). From the Table 2(see PDF) it was found that, MDA level raised significantly for breast cancer patients without treatment 4.63±0.053 and after treatment 6.56±0.169 when compared to normal control subjects 1.9±0.1. On the other hand, AC-T chemotherapy induces an increase in the lipid peroxidation compared to healthy controls. The Mean value of TAS level was higher for control group 163.36± 8.84 than breast cancer patients without treatment 129.96±10.53 and with treatment 97.27±7.6. The serum NO increased in breast cancer patients after chemotherapy 66.1±1.23 and before chemotherapy 54.6±1.79 as compared to normal control25.73±1.14 (Table 3 - see PDF). AC-T chemotherapy caused an increase in NO compared to patients before treatment and to healthy controls. Serum antioxidants catalase activity and the level of TAS were significantly diminished in breast carcinoma patients before treatment as compared to normal control. These changes more declined with AC-T chemotherapy as compared to breast cancer patients and healthy controls (Table 2 - see PDF). The liver enzymes ALT and AST activities andrenal creatinine and blood urea levels show change in patients before treatment or after receiving AC-T in comparison to healthy control (Table 3 - see PDF). Figure 1 a-c(see PDF) represented the various clinical images of mammograms. Figure 2 a-c(see PDF) showed the Photomicrograph showing strong nuclear estrogen receptor (ER), strong nuclear progesterone receptor (PR) and uniform intense membrane HER2/neu immune reactivity in tumor cells.

## Discussion:

In breast cell, chemotherapy causes an increase in free radical generation, which leads to decline in the antioxidant content, resulting in oxidative stress [27]. During cancer chemotherapy, cytotoxic medicines will able to yield highly reactive free radicals, which operate as collective apoptosis mediators [28, 29, 30]. The excess production of reactive free radicals or the exhaustion of antioxidants is denoted as oxidative stress [31]. Excessive synthesis of OFR can damage biomolecules, leading to lipid peroxidation, mutagenesis, and carcinogenesis. OFR-induced lipid Peroxidation is associated in neoplastic transformation. OFR can cause fibroblast proliferation, epithelial hyperplasia, cellular atypia, and breast cancer in the breast epithelium [32]. Look and Musch [33] reported that, in cancer patients the antioxidant capacity was significantly reduced after repeated poly chemotherapy with radical-generating chemicals, resulting in oxidative stress. Increased cytotoxic activity in breast cancer patients may be due to high levels of TBARS and decreased TAS in chemotherapy. As a result, controlling MDA and production of TAS is critical for the breast carcinoma treatment. When the data of the present study was analyzed at other stages, II and III, a substantial variance was identified in MDA levels between the 2 groups before and after treatment. Furthermore, the amount of TAS in these patients was significantly reduced [33]. The MDA serum concentration was substantially higher after therapy (p=0.05) than before treatment and controls in the current study. Because MDA is a lipid peroxidation surrogate marker, it plays a prominent role in breast carcinoma development. Our findings matched those of Rajneesh et al. (2008) [32]. TAS levels were found to be significantly lower after treatment than before treatment. NO is a crucial molecule involved in a variety of physiological processes. NO is a highly reactive free radical in a biological systems that combines with other free radicals, molecular oxygen, and heavy metals. Peroxynitrite (ONOO-) and N2O3 are produced by NO, which can cause DNA damage. Peroxynitrite has the ability to oxidise and nitrate DNA, as well as produce single-strand DNA breakage by attacking the sugar phosphate backbone. NO levels have been linked to the promotion or inhibition of cancer genesis in a number of studies [33- 34]. Breast cancer and chemotherapy can cause a lot of NO generation, which can lead to a lot of cytotoxic activity. Hence, the mechanism that prevents NO production is critical for breast cancer chemotherapy. NO activates oncogenic signaling pathways such as extracellular signal-regulated kinases (ERKs) and phosphoinositide 3-kinases (PI3Ks) to promote cancer progression [35]. NO levels were substantially higher (p >0.05) after treatment than before treatment and controls in the current investigation. Catalase activity in breast cancer patients was shown to be considerably lower after treatment than before treatment in our study. These findings are consistent with those of Prabasheela et al. [36] and Kasapovi et al. [18], who reported that catalase and superoxide dismutase activity decreased in breast cancer patients, indicating increased free radical activity while the antioxidant defence system was reduced. Most animal and human malignancies produce ROS and have low amounts of antioxidant enzyme biochemically. The mean serum urea and creatinine levels in patients earlier to the 1stcycle of chemotherapy and those in patients subsequently the 3rd cycle of chemotherapy was not statistically different in the present study. These findings were reliable with Noviyani et al. findings from Indonesia [37]. In present study renal function tests of blood urea, Serum creatinine were significantly differ (p >0.05) in after treatment than before treatment than controls. These chemotherapeutic medications are hydrophilic, so they can't get pass the cell's inner membrane, and can be reduced by NADH on the surface [38, 39]. Chemotherapeutic medicines, such as doxorubicin, which is used to treat AC, can pass through the outer mitochondrial membrane and into the cytosol. Intramolecular rearrangements lead to the creation of a lipophilic deoxyaglycone that can pass through the mitochondrial inner membrane. As an electron acceptor, doxorubicin competes with coenzyme Q10 and averts electrons to molecular 02, resultant in the creation of super oxide radicals [39]. DOX blocks the DNA and RNA synthesis, hindering the cell cycle phase, and triggering apoptosis of tumour cells in the G2 phase by blocking the cell cycle and inhibiting the DNA polymerase enzyme in tumour cells by DNA intercalation and inhibition of topoisomerase II. The production of free radicals can cause DNA to break, adding to an increase in oxidative stress42 caused by a decrease in the availability of other antioxidant endogens [40, 41]. Our findingsshowed that AC-T therapy increased liver enzymes AST and ALT (p <0.05), indicating that serum levels of GOT and GPT are often utilized as hepatic damage indicators because intracellular enzymes are released into the blood after hepatic injury [42]. Comparisons of mean serum GOT levels were not significant in this study. Breast cancer patients' baseline serum GPT levels, on the other hand, were considerably greater than controls (P = 0.02). The levels of serum GPT measured after the third cycle of chemotherapy were greater than those measured before the first treatment, although the difference was not significant. Some studies found a significant increase in serum levels of SGOT and SGPT (p <0.05) in breast cancer patients after treatment [43]. Finally, monitoring serum oxidative stress indicators may aid in the evaluation of chemotherapy effects in breast cancer patients. Chemotherapy with AC-T elevated malondialdehyde, a lipid peroxidation marker, and lowered total antioxidant status, according to our findings. Increased NO levels and lower catalase levels in breast cancer patients indicate oxidative stress. Because AC-T chemotherapy increases oxidative stress, which can lead to hepatotoxicity and cardiotoxicity, it's important to keep track of serum oxidative markers and liver enzymes. The findings suggested that oxidative stress and the development of hepatotoxicity may promote breast cancer growth, presumably via catalase, TAS, MDA, NO, AST, and ALT activities.

## Financial support:

The study was not supported by any grants and funds.
